# Frequency of success and complications of primary endoscopic third ventriculostomy in infants with obstructive hydrocephalous

**DOI:** 10.12669/pjms.38.1.4097

**Published:** 2022

**Authors:** Seema Sharafat, Zahid Khan, Farooq Azam, Mumtaz Ali

**Affiliations:** 1 Seema Sharafat, FCPS. Department of Neurosurgery Medical and Teaching Institute, Lady Reading Hospital, Peshawar, Pakistan; 2 Zahid Khan, FCPS. Department of Neurosurgery Medical and Teaching Institute, Lady Reading Hospital, Peshawar, Pakistan; 3 Farooq Azam FCPS. Department of Neurosurgery Medical and Teaching Institute, Lady Reading Hospital, Peshawar, Pakistan; 4 Mumtaz Ali, FCPS. Department of Neurosurgery Medical and Teaching Institute, Lady Reading Hospital, Peshawar, Pakistan

**Keywords:** Congenital Hydrocephalus, Endoscopic Third Ventri-Culostomy, Infants, Obstructive Hydrocephalus, Ventriculo-Peritoneal Shunt

## Abstract

**Objectives:**

To determine the success rate and complications of primary endoscopic third ventri-culostomy (ETV) in infants with obstructive hydrocephalous.

**Methods:**

This case series was conducted at the Department of Neurosurgery, Medical and Teaching Institute, Lady Reading Hospital Peshawar from July 2016 to June 2018. All consecutive patients with age less than one year who underwent ETV for primary obstructive hydrocephalous, of both gender, were included in the study. The patients were followed up to six months after surgery. The data was entered in a specially designed Performa. Patients’ data was analyzed using SPSS version 21.0.

**Results:**

We had total 21 patients with age less than one year during the study period. Male patients were 11 (52.4%). Success rate of ETV at six months of follow up was 12 (57.1%). Post-op complications observed were in 9.52% (2/21) cases. One patient had cerebrospinal fluid CSF) leak and the other had significant bleed.

**Conclusion:**

ETV is successful in 57.1% of infants with obstructive type of hydrocephalous. The post op complications in case of ETV are lower than Ventriculo-peritoneal shunts. Therefore, ETV can be offered to infants having obstructive hydrocephalous.

## INTRODUCTION

Hydrocephalous is excessive accumulation of cerebrospinal fluid in the cranium and ventricular system causing increase intracranial pressure.^1^,^2^ Hydrocephalous can be treated both medically and surgically. Most of the infants with hydrocephalous are treated surgically and Ventriculo-peritoneal shunt (VP shunt) is the standard treatment option. Ventriculo-peritoneal (VP) shunt is for all types of hydrocephalous. However, it is having high failure rate because of malfunction and infection.^2^,^3^

The alternative treatment option is endoscopic third ventri-culostomy (ETV).^2^ ETV was performed successfully by Mixter et al. in 1923.^4^ Since then it is mainly indicated in patients with obstructive hydrocephalous when the obstruction is at or behind the posterior half of the 3rd ventricle. So, the common indications of ETV are aquiductal stenosis, pineal tumors and tectal gliomas.^5^ Some studies have reported that ETV is also helpful in post infection hydrocephalous.^6^

ETV is a convenient and easy mode of hydrocephalous treatment. In ETV, we by-pass the site of obstruction and the CSF is directed to flow from the third ventricle to the inter-peduncular and pre-pontine cisterns.^1^,^3^ As there is limited local study on the ETV success and complications in infants, this study will determine the frequency of success rate and complication of primary ETV in infants with obstructive hydrocephalous.

## METHODS

This case series was conducted at the department of neurosurgery, medical and teaching institute (MTI) lady reading hospital Peshawar for a period of two years from July 2016 to June 2018 after approval from institutional ethical review committee (Ref No# 227/LRH, dated: October 28, 2019). All consecutive patients with age less than one year who underwent ETV for primary obstructive hydrocephalous, of both gender, were included in the study after written informed consent from parents or guardians of the patients. Patients with age more than one year, previous surgery for hydrocephalous (ventriculo-peritoneal shunt or ETV) and hydrocephalous associated with congenital neurospinal disorders were excluded from the six months after surgery to observe success and complications. Complication will be treated accordingly. Data was collected on a pre-designed performa and analyzed using SPSS version 21.0. Frequency and percentage was categorical variables.

## RESULTS

A total of 21 patients with age ranged from 0-1 year (infants). Most (11/21, 52.4%) of our patients were male as given in Table-I. Success rate of ETV at six months of follow up was in 12 (57.1%). The rest were subjected to Ventriculo-peritoneal shunts after ETV failure. Post-operative complications were observed in 2 (9.52%) cases. One patient had cerebrospinal fluid CSF) leak and the other had significant bleed. The patient with CSF leak responded to conservative treatment while the one with bleed were passed external ventricular drain (EVD) later on converted to VP shunt. None of our patients had operative mortality.

**Table-I:** Gender distribution of infants with hydrocephalous (n=21).

Gender	Frequency	Percentage
Male	11	52.4
Female	10	47.6

**Table-II:** Efficacy and Post-operative complications.

Variables	Frequency	Percentage
Efficacy	12	57.1
Total complications	02	9.52
Bleeding	01	4.76
CSF leak	01	4.76

## DISCUSSION

Endoscopic third ventriculostomy (ETV) is comparatively an easy way of treating hydrocephalous.^7^ The success of ETV depend upon the age of the patient, cause of obstruction and previous shunting surgery. Results are different regarding success of endoscopic third ventriculostomy in different age groups. Most of the studies have reported that it is more (88%) successful in adults than in infants with age less than 6 months.^8^ Woodworth et al. reported in their study that ETV was successful in 55% of adults.^9^ In almost similar study, Dusick et al. reported that it was successful in 77% of adult patients with hydrocephalous.^8^ The possible explanation of less successful ETV in infants may be because of less absorption of CSF from the arachnoid villi and early closure of the stoma due to formation of new arachnoid membranes.^10^ The other reason of ETV failure in infants may be because the fontanels and cranial sutures are open and the intraventricular pressure is not sufficient to push the CSF across the newly formed stoma to the interpeduncular cisterns.^9^,^10^

However, the concept that ETV is less successful in infants and children is not always true. Some of the studies have reported that ETV is also successful in children with obstructive hydrocephalous. They have reported that it is not the age but the cause of hydrocephalous which affect the success of ETV.^11^ In our study we observed that ETV was successful in 57.1 % of the infants. In a study 1406 children who undergone Endoscopic third ventriculostomy, the success rate was 55.6%.^12^ Baldauf et al., reported in their study that ETV success is 37.1% of infants with age less than 1 year.^13^ Fritsch and colleagues reported that ETV success is about in 39 % of infants and the age should not be contradiction of ETV.^14^ The overall success rate of ETV in patients with the obstructive hydrocephalus is between 50 and 94%.^14^

Like other surgical procedures ETV has its own complications. On literature review post op complications after endoscopic third ventriculostomy ranges from 0-31.2%.^15^ The common complications are hemorrhage, infection (1.8-6.1%), subdural collection, injury to thalamus/ hypothalamus (0.5-1.4%) and cerebrospinal fluid leak (1.7-5.2%).^15^,^16^ In our study we observed that post op complications occurred in 9.52% (2/21) cases. One of the patients had bleeding due to minor vessel injury. That patient needed external ventricular drain (EVD) which was later on converted in to VP shunt. The other one was having CSF leak however it was treated conservatively. Our results can be compared to the work done by other people. It has been reported that almost 8.5% patients get complicated after endoscopic third ventriculostomy.^15^,^17^ In one study of 89 patients the post op complications after ETV were 7.9% (7/89). All of these complications were transient. ETV failure occurred in 32.6% cases.^18^ In a local study of 45 patients who underwent ETV for the treatment of obstructive hydrocephalus., the overall complication rate was 17.8% (8/45) and the most common (13.3%) complication reported in the study was infection followed by hemorrhage (8.9%).^19^ Most (16.5%) of the bleeding during ETV are minor and are treated usually with irrigation. However fatal bleeding due to basilar artery injury occurs in 0.49% cases.^15^-^17^ Once endoscopic third ventriculostomy (ETV) is successful it avoid shunt dependency which have high risk of complications and failure.^20^ If we compare our results with the one with VP shunts we see that almost 30-40 % of all shunts in children fail within 1 year of placement.^21^ In another study of 128 children with hydrocephalous VP shunt failure was observed in 52.4% cases and shunt infection was seen in 23.4% cases.^22^ Others have reported that post op shunt infection occurs in 5-15% cases.^23^ So here our results are much better. The overall complications after VP shunt is in the range of 20-80%.^24^ The published mortality after ventriculoperitoneal shunt is almost 5%.^25^ However, none of our patients had fatal out come during the study period. So here again or results are better than VP shunts.

### Limitations of the study:

There are certain limitations that need to be considered. The first and provost among them was our inability to include the neuroimaging records of the patients included with obstructive hydrocephalus. Although, the sample size was small but it was a strong representation of the neurology patients treated at a single-center.

## CONCLUSION

We conclude from our study that the success rate of endoscopic third ventriculostomy (ETV) is 57.1% of infants with obstructive type of hydrocephalous and the frequency of complications are 9.52%. The post op complications in case of ETV are lower than Ventriculo-peritoneal shunts. Therefore, endoscopic third Ventriculo-ostomy can be offered to infants having obstructive hydrocephalous.
